# Correction: When ELIZA meets therapists: A Turing test for the heart and mind

**DOI:** 10.1371/journal.pmen.0000426

**Published:** 2025-08-29

**Authors:** S. Gabe Hatch, Zachary T. Goodman, Laura Vowels, H. Dorian Hatch, Alyssa L. Brown, Shayna Guttman, Yunying Le, Benjamin Bailey, Russell J. Bailey, Charlotte R. Esplin, Steven M. Harris, D. Payton Holt, Merranda McLaughlin, Patrick O’Connell, Karen Rothman, Lane Ritchie, D. Nicholas Top, Scott R. Braithwaite

The Funding statement is updated to:

This study was supported by internal funds to author SRB from the Department of Psychology, Brigham Young University. The content is solely the responsibility of the authors and does not necessarily represent the official views of the Brigham Young University.

The Competing interests statement is updated to:

At the time of publication, SGH is owner of Hatch Data and Mental Health, where he offers therapy and conducts research. The website can be accessed here and was not available at the time of publication: hatchdamh.com. SGH receives financial compensation from seeing clients through Hatch Data and Mental Health. None of these clients are represented in the publication. Hatch Data and Mental Health did not fund this study. ZTG and HDH do not hold financial interests in Hatch Data and Mental Health, nor do they receive any financial compensation from or with the affiliation. As this is a therapy business, we do not carry titles as Hatch Data and Mental Health is less of a corporate ladder and more of a feelings-forward free climb.
