# Supplementary material for: When ELIZA meets therapists: A Turing test for the heart and mind
Source: PLOS Ment Health. 2025 Feb 12;2(2):e0000145. doi: 10.1371/journal.pmen.0000145 (PMC12798195; doi:10.1371/journal.pmen.0000145)
Supplement: S1 Table — (DOCX) [file pmen.0000145.s001.docx]

**S1 Table**

Vignettes and appropriate authors.

| **Vignette 1:** A couple gets referred to you for treatment by a colleague. You offer a free initial consultation. The couple asks: “Why do you think you’d be able to help us?” How would you respond? | |
| --- | --- |
| **Therapist:** That is a great question! This is actually what we are all going to collaboratively explore today in this consultation. I would love to get a better idea of the current difficulties you both are facing, what you have tried before, your thoughts on trying psychotherapy, as well as find out what IS working. After that, I can give you both an idea of how psychotherapy can help. I will bring up approaches that map onto your current concerns and discuss together what those next steps could be. Does this sound okay to you both as a place to start? | **ChatGPT:** I appreciate your question and the thoughtfulness behind it. It's crucial to feel a sense of trust and confidence with your therapist. In my approach to couple's therapy, I prioritize creating a safe, neutral space where both of you can express yourselves openly and feel heard. My goal is to understand each of you individually, as well as your dynamic as a couple, and to work collaboratively with you to address the challenges you're facing. My practice is rooted in evidence-based therapeutic approaches, including Emotionally-Focused Couple Therapy and Behavioral Couple Therapy, which have been proven to help couples improve communication, deepen emotional connections, and resolve conflicts effectively. However, I also understand that therapy is not one-size-fits-all. I tailor my approach to meet the unique needs and circumstances of each couple, taking into account your personal histories, cultural backgrounds, and the specific issues you're facing. Empathy is a cornerstone of my work. I strive to empathize with both of you, recognizing and validating each of your feelings and experiences. It's important that neither of you feels sidelined or misunderstood in this process. In terms of professionalism, I adhere to the ethical standards set by the APA and AMA, ensuring that our work together is conducted with integrity, confidentiality, and respect for your autonomy and well-being. Cultural competence is also integral to my practice. I am committed to understanding and respecting the cultural nuances that shape your experiences and perspectives. This involves an ongoing effort to educate myself and to approach each session with cultural sensitivity and openness. Ultimately, my role is to help you find new ways to understand and support each other, building a stronger, more resilient partnership. It's a journey that requires commitment and work from all of us, and I am here to guide and support you through this process. |
| **Vignette 2:** After only three sessions, a couple you are seeing continues to struggle with the same argument that brought them into couple therapy. Out of frustration, one partner mentions: “So, when are we supposed to get better? This is taking a long time.” How would you respond? | |
| **Therapist:** Frustrated Partner, thank you for bringing this up, because you are absolutely right, this is taking a long time! I imagine this conversation was frustrating for you and for Other Partner. One thing is you both have identified this argument as one of your main concerns in previous sessions and it sounds like you both are committed to moving through this issue. However, this problem has been going on for quite a while and not only is it difficult to shift these patterns, but I, as a therapist am still getting to know both of you. You both have shown to be incredible problem solvers and bright, and if this required a simple solution, I am absolutely certain you both would have solved it. So, to answer your question, it may take us a few more sessions to find a solution that is the right fit. However, the fact that you are trying and wanting things to improve is a great sign; in fact I think it might be helpful for us to review what happened just now for each of you. I think this would give us insight into why this argument emerged despite both of your efforts. What do you think? | **ChatGPT:** I hear your frustration and the sense of urgency for change. It's natural to want to see immediate results, especially when you're investing time and emotional energy into therapy. Remember, meaningful and lasting change often takes time and can be a gradual process. It's like nurturing a plant; the growth isn't always visible day to day, but over time, the changes become more apparent.  Let's acknowledge the steps you've both taken so far. Being here, openly discussing your challenges, and committing to work through them are significant achievements. Sometimes, the progress in therapy can be subtle and may not be immediately evident. It might be helpful to explore what 'getting better' means for each of you. This can give us a clearer direction and help set realistic expectations for our sessions.  I'm committed to supporting both of you through this journey. It's important for us to maintain an open dialogue about your therapy experience, including any concerns or frustrations you might have. This feedback is valuable and helps me adjust our approach to better suit your needs. |
| **Vignette 3:** A couple comes to session reporting difficulties in navigating household responsibilities. When you ask them to explain more about this issue, one partner responds, “Well, I’m really clean and tidy and they’re absolutely disgusting. The other day, they didn’t even wash their dish after dinner.” The other retorts, “It wasn’t a dish, it was a fork, and I was rinsing it but you asked me to take out the trash – which I did. I’m not even that messy, you’re just a clean freak!” How would you respond? | |
| **Therapist:** I'm hearing a lot of frustration about this issue, but I'm also hearing each of you characterizing your partner as the problem, rather than the problem as the problem. Can we take a step back and think about the impact of using language like "absolutely disgusting" or "clean freak?" Looking past the person, what is really bothering you here? | **ChatGPT:** I hear that cleanliness and tidiness are very important to you. It can be frustrating when it feels like your standards aren't being met, especially in your own home. It's clear that you value a certain level of order and cleanliness.  And it sounds like you feel your efforts are being overlooked and that the expectations around cleanliness might feel a bit overwhelming. It's important that your efforts, like taking out the trash, are recognized and that there's a balance in how household tasks are approached.  In relationships, differing perspectives on household responsibilities are common, and finding a middle ground that respects both of your needs is key. How about we explore what a fair and mutually satisfying arrangement might look like? This could involve setting clear and reasonable expectations and finding ways to appreciate each other's contributions to maintaining the home. |
| **Vignette 4:** You’ve struggled for the last little while with a couple and feel you are spinning your wheels. Despite your best efforts, therapy appears to be non-specific and difficult to disentangle. You bring this up to the couple within session. Nervously, the couple looks at one another and one eventually responds, “Well, we’ve really been struggling with, well, intimacy.” How would you respond? | |
| **Therapist:** Thank you for sharing and I recognize that many people use the word intimacy in different ways. Emotional intimacy is at the root of many of these, and sometimes people are using the word to talk about their sex life as a couple. Can you help me understand more what we mean here? | **ChatGPT:** First, I want to acknowledge the courage it takes to bring up something as personal as struggles with intimacy. It's not an easy topic to discuss, and I appreciate your willingness to share this with me. Recognizing and addressing challenges in this area is a significant step in working towards a healthier, more fulfilling relationship.  Intimacy issues are quite common in relationships and can stem from a variety of sources. It's important to understand that these challenges don't diminish the value of your relationship or your connection with each other. Instead, they offer us an opportunity to explore deeper aspects of your relationship and individual experiences that might be influencing your intimacy.  In our sessions together, we can work on creating a safe space where both of you feel heard and understood. It's crucial that we approach this topic with sensitivity and without judgment. We'll aim to understand each other's perspectives and feelings, and I'll help guide you through this process with empathy and respect for both of your experiences.  We can explore various aspects of intimacy - emotional, physical, and psychological - and identify areas where you both feel comfortable and areas where you may need more support. Our goal will be to enhance communication, increase emotional connection, and find ways to comfortably express your needs and desires to each other.  Remember, building intimacy is a journey, and it often involves navigating complex emotions and vulnerabilities. It's okay if things don't change overnight. What's important is that we're taking these steps together, and I'm here to support you both through this process. |
| **Vignette 5:** As you are in the process of gathering information for a new case, you ask the couple what their goals might be for therapy. One partner looks at their partner, and back and you, and mentions, “Well, I guess we would like to improve our communication skills.” How would you respond? | |
| **Therapist:** Fantastic, improving communication skills is definitely a goal we can work toward. That can mean a lot of different things, to different people and in different situations. I'd like to hear from each of you about what parts of communication have been difficult, and also how you'd like for communication to go between the two of you. Let's say in a couple of months communication skills were improved, how would we know? What would the two of you be doing more--or less--of?  [After that topic has concluded, address the partner who had looked to the other before answering:] [(If true) It sounds like improving communication skills is a goal that both of you have.]  [Name], what's another goal that you have for therapy? | **ChatGPT:** Improving communication is a common and important goal in couple's therapy. It's great to hear you both agree on this. To better understand your needs, could you each describe what effective communication looks like in your relationship? Are there specific scenarios where you feel your communication could be stronger, or perhaps times when it feels particularly successful? This insight will help us focus our efforts more effectively, ensuring that we address the areas most important to you both as a couple. |
| **Vignette 6:** An unmarried couple seeks your assistance in preparation for becoming married - an intervention recommended by their local pastor. As you’re assessing the couple’s goals for therapy, one partner shares, “My parents were divorced and I don’t want the same thing to happen to us. What can we do now to avoid that? I don’t want our kids to go through what I went through!” How would you respond? | |
| **Therapist:** That's really helpful to acknowledge, the fears we come into a relationship with, wanting to be like or not be like our parents in their relationships. I'd like to talk more about the fear of divorce in particular. Sometimes this fear can keep us from really engaging with conflict in helpful ways, since when we do our fear "don't let this lead to divorce!" shuts us down and we can get stuck as a couple. Instead, it can help to remind yourself that working through conflict is key to healthy relationships, that divorce is a choice that isn't preferred, but the main idea is to promote a healthier relationship THROUGH working through conflict rather than needing to always avoid divorce at any cost. For many people, it's more helpful to consider the cases when divorce WOULD be on the table, helping them recognize that a mundane conflict really isn't one of those situations. | **ChatGPT:** Thank you for sharing your concerns and goals for therapy. It's commendable that you're both taking proactive steps to build a strong foundation for your marriage. The desire to avoid the pain of divorce, especially considering your personal experience, is completely understandable. It's important to acknowledge that the fear of repeating your parents' experience is valid and significant.  In addressing your concern, we can explore several approaches. For instance, Emotionally-Focused Couple Therapy (EFCT) can help you both understand and respond to each other's deeper emotional needs, fostering a stronger bond. This approach is particularly effective in helping couples build resilience against stressors that might challenge a marriage.  Additionally, incorporating elements from Integrative Behavioral Couple Therapy (IBCT) can help you both accept differences that are not easily changeable, promoting a deeper understanding and appreciation of each other. This acceptance is crucial in navigating the inevitable challenges that arise in any marriage.  Moreover, it might be beneficial to discuss and set clear, mutual goals for your relationship and future family. This process involves open communication about expectations, fears, and desires, which can be facilitated in a safe and supportive environment.  Remember, every couple's journey is unique, and it's important to tailor our approach to fit your specific needs and cultural backgrounds. Your proactive attitude towards building a healthy relationship is a strong foundation to work on. Let's explore these options together and find the best path forward for both of you. |
| **Vignette 7:** A lesbian couple that has been together for 20-years has started therapy with you - it was clear in their intake paperwork they really wanted to focus on their ability to coparent. During the assessment phase of therapy, you typically interview both partners together and then separately to gauge their perception of the goals they have for therapy. During the joint session, it appears that the couple is struggling to parent their teenage son who they recently discovered is using drugs. One partner is more involved insisting their son needs to attend therapy to get this problem sorted out. The other partner, though not condoning of the drug use, takes a more empathic approach suggesting they forgive the child and move on. In the individual sessions, the more permissive partner (i.e., forgive the child) discloses tearfully the following, “I had an affair at the beginning of our relationship while we were dating. I’ve felt tremendously guilty and have never forgiven myself. Should I tell her? Do you think this is impacting our ability to coparent?” How would you respond? | |
| **Therapist:** I'm sorry you're having this experience, it can be very hard, and isolating, to carry that kind of guilt. I can't say how you should best handle sharing this information, or the extent to which it might be affecting your ability to coparent. You know your partner much better than I do, so what utility do you see in sharing? Or more specifically, why do you think this is now on your mind after twenty years? How do you think it might be impacting your ability to make parenting decisions? | **ChatGPT:** It's clear that you've been carrying a heavy burden with this secret and it's impacting you deeply. The struggle you're facing about whether to disclose the affair to your partner is a profound one, and it's understandable that you're considering its influence on your current family dynamics, especially regarding co-parenting.    In our sessions, we can explore the depth of your guilt and how it may be influencing your approach towards your son and your relationship with your partner. This exploration will help in understanding the complexities of your emotions and how they intersect with your family life.  The decision to share this information with your partner is a significant one and should be approached with care and consideration of all possible outcomes. It's important to reflect on how this might affect your partner, your relationship, and the overall family dynamic.  We can also work on strategies for managing these difficult emotions and improving communication within your relationship. Developing these skills can be beneficial, regardless of whether you choose to disclose the affair or not.  My role is to provide a supportive and non-judgmental space for you to process these feelings and to assist you in making decisions that are in the best interest of you and your family. |
| **Vignette 8:** A mixed-race straight couple (husband is Black and wife is White) present to therapy mentioning they have difficulties coping with in-laws. During a family party, the husband heard the wife’s brother utter a racial slur. The husband was very hurt, deeply angry, and said to his wife that he never wanted to return to any of the family events. The wife mentions, “My brother is an idiot and his behavior was unacceptable; however, I don’t understand why this means we can’t visit anyone in my family anymore.” How would you respond? | |
| **Therapist:** Wife's Name, I am glad to hear that you recognize how unacceptable this behavior is. It sounds like you care deeply for your husband and do not want him to be hurt. I also hear confusion. When you experience this confusion with your partner, this is an excellent opportunity to explore the situation from his perspective. I want you to put yourself in your husband's shoes for a bit here. Husband's name, can you review some of the emotions you felt in the moment?  Thank you. Wife's Name, I want you to sit with those emotions and imagine what you might feel entering that space again. What emotions or worries might you have in that moment? | **ChatGPT:** To the husband: "I hear the pain and anger in your words, and it's important that we recognize the deep impact of what you've experienced. Your feelings in this situation are valid and deserve attention. Could you share more about how this has affected you and what you need to feel supported in this relationship?"  To the wife: "Your acknowledgment of the wrongdoing and support for your husband is clear, yet I sense this situation is also challenging for you. Your perspective is crucial here. Can you express how you're feeling about this and how we might work together to address the needs of both you and your husband?" |
| **Vignette 9:** A gay couple present to treatment mentioning struggling in their comfort with outness. Tom, who is open and extroverted, feels very comfortable holding hands, hugging, and kissing in public; however, Joe, more introverted, grew up with a conservative Christian background, and feels uncomfortable with these “grand displays of affection.” Joe describes, “No one needs to see us kissing in public. You know I love you, and I know you love me. I’ll kiss you all you want once we’re at home.” How would you respond? | |
| **Therapist:** This is a really common issue for couples - sorting out what it looks like to be together in different contexts. It sounds like there are lots of moving pieces, for each of you, in terms of what it means to be affectionate with each other in public. Tom, it sounds like, for you, holding hands, hugging, and kissing in public are ways to communicate how you feel about each other. Is that right? I am wondering how you interpret Joe's hesitation here. What does it mean to you or about your relationship, in your mind?  And, Joe, it sounds like you feel uncomfortable holding hands, hugging, and kissing in public, is that right? Can you tell us a little bit more about this discomfort? Is it the same level of discomfort for all affectionate behaviors in public? Is it the same level of discomfort in all ways of being public? In other words, are there factors like who is around or geographical differences that make it more or less comfortable? | **ChatGPT:** Tom, Joe, thank you both for sharing your feelings so openly. It's clear you both deeply value your relationship and have different comfort levels when it comes to expressing affection in public. Tom, your ease with public displays of affection reflects your open nature, and Joe, your preference for more privacy is equally understandable, given your background.  In relationships, respecting each other's comfort levels and finding a balance that works for both partners is important. Let's explore what forms of affection you are both comfortable with. It might be helpful to consider small steps that respect both of your boundaries while allowing for expressions of affection that feel right for both of you.  Additionally, understanding the emotions and beliefs behind your preferences can be valuable. This understanding can help build empathy and deepen your connection. How does this sound to both of you? Would you be open to discussing this further in our sessions? |
| **Vignette 10:** A straight couple comes to your office with a considerable age difference (husband age 76; wife age 32) and were just married after the husband’s wife recently passed away. The husbands children (ages 45, 48, and 53) disapprove of the marriage and no longer visit the gentleman after he made his new wife part of his estate. Both the husband and wife feel heartbroken, and the wife mentions: “I don’t want to come between him and his children. I’ve asked to be removed from the estate and I don’t care about the money - I have a successful business on my own. I sincerely love him, care about him, and want to spend the rest of our lives together.” How would you respond? | |
| **Therapist:** Of course. It sounds like asking to be removed was a way to prove this to his kids. I am curious about this idea that you, [wife], have come between him and his children. [Husband], can you tell me more about your relationship with your kids and how that has changed over time? | **ChatGPT:** I can see how much this situation is impacting both of you. It's clear that you both value your relationship deeply and are facing these challenges as a united front.  To the wife, your decision to potentially remove yourself from the estate is a significant gesture that underscores your dedication to your husband and your relationship, rather than material benefits.  To the husband, coping with the distancing from your children during this new chapter in your life must be deeply distressing, especially following the loss of your previous wife.  Have you considered ways in which you might approach your children to discuss these issues? Open and heartfelt communication about your feelings, your commitment to each other, and the respect for your family's emotions might be a crucial step forward.  It's important to remember that you're in this together. Your mutual support and understanding are vital as you navigate these difficult family dynamics and work towards finding a resolution that honors your relationship and family connections. |
| **Vignette 11:** A straight couple comes presents to treatment after the husband’s parents were killed in a car crash two year previously. The husband is dealing with symptoms consistent with Post Traumatic Stress Disorder, and his wife responds: “He’s so depressed all the time. Like, I understand, his parents were killed. However, he’s got to go to work, show up for his kids, and get out of bed in the morning.” How would you respond? | |
| **Therapist:** I am hearing that Husband is experiencing significant mental health concerns that are interfering with his work and family obligations. I am sensing concern for Husband and for his wellbeing, as well as some frustration at the extra responsibility you have had to take on in response to his mental health concerns. I am curious about what Husband is thinking or feeling about that statement. I can imagine myself having mixed feelings about that statement if I was him. Husband, do you mind sharing your experience? | **ChatGPT:** I hear how challenging this situation has been for both of you. [Husband's name], the loss of your parents in such a tragic way is a profound and life-altering event. The symptoms you're experiencing, which sound like they could be related to Post Traumatic Stress Disorder, are a natural response to such a significant loss. It's understandable that this has deeply affected your mood and daily functioning.  At the same time, [Wife's name], it's clear that you're facing your own challenges in this situation. Your feelings of wanting to see [Husband's name] engaging in life more actively and being present for work and family are valid. It's tough to see someone you love struggling and to feel the impact on your family and daily life.  In therapy, we can work together to address both of your needs. For [Husband's name], we can explore ways to help you process your grief and manage your symptoms in a healthy way. For [Wife's name], we can look into support strategies for you as a partner and discuss how you can both communicate effectively during this difficult time. It's important to remember that healing is a process, and it requires patience and understanding from both sides.  I'm here to support both of you through this journey, respecting your individual experiences and working collaboratively to find a path forward that acknowledges and addresses both of your needs. |
| **Vignette 12:** You’re working with an especially argumentative couple. At one point, one partner (the more aggressive one) turns to you and mentions, “What’s the point of couple therapy if you are just going to sit on the sideline and watch us argue?” How would you respond? | |
| **Therapist:** That's a great question. Actually, what I'm doing is watching and listening for patterns so that I can better understand why conversations keep going off the rails for the two of you. What is it like for you when I sit here observing? | **ChatGPT:** I understand your frustration and why it might seem like I'm on the sidelines. It's important for me to observe your interactions to better understand the dynamics between you both. However, my role isn't just to observe. It's also to guide and support you in finding more constructive ways to communicate and resolve conflicts. It seems like the way arguments are happening now is causing both of you distress. Let's work together to explore different approaches. I'm here to help you both feel heard and understood, not just to witness your arguments. How does that sound to you both? |
| **Vignette 13:** You normally have a sliding scale fee (e.g., half price) for a few couples that are low-income. You recently started with a new sliding scale straight couple that can only see you once every other week because of the expense. The husband is returning to school after a decade away while the wife is staying home with their four children. After reducing your fee further, and meeting with the couple for six sessions, a clear pattern emerges: their relationship distress is caused due to persistent financial difficulties. The wife mentions: “We love each other dearly; however, this financial stress is crushing us. We have literally tried everything but we have been rejected from government welfare programs because we make a little too much money and inflation is making it all worse. How are we supposed to do this on our own?” You look to the husband to see him nod in helpless agreement. How would you respond? | |
| Therapist: It's hard to meet not just our needs, but the needs of a relationship, when under financial stress. I'm sorry you're going through this difficult time. It is easy to let a relationship take a back seat when finances are strapped and we feel like we are just focused on the basics, trying to survive. But it doesn't make the needs of the relationship disappear. If we can step back for a minute, what are one or two things that have taken a back seat recently? | ChatGPT: Thank you for sharing your feelings so openly. It's clear that you both are facing a significant challenge with the financial stress, and it's affecting your relationship deeply. It's important to recognize the effort and commitment you both are putting into your family and each other, despite these hardships.    Let's focus on how we can strengthen your partnership in the face of this stress. Even though we can't change the external financial situation in our sessions, we can explore strategies to enhance your emotional support for each other. For instance, we can use techniques from Emotionally-Focused Couple Therapy to help you both express your fears and needs in a way that brings you closer, rather than adding to the stress.  Additionally, it might be helpful to look at your situation through the lens of Cognitive Behavioral Couple Therapy, identifying any negative thought patterns that could be contributing to your stress and learning ways to counteract them. This approach can help in improving communication and reducing feelings of helplessness.  I would also like to provide you with some additional resources outside of our sessions. There might be community programs, financial counseling services, or support groups that could offer some assistance or guidance. While these resources won't solve all the challenges, they may provide some relief or new avenues to explore.  Remember, your relationship's strength lies in how you support and understand each other, especially during these tough times. You're not alone in this; we're here to work through this together, finding ways to lighten the emotional burden and strengthen the bond you share. |
| **Vignette 14:** A husband finds out his wife has recently engaged in an affair. The wife minimizes it by saying, “I didn’t have an emotional connection - it was purely physical.” To which the husband responds: “How stupid are you? You clearly have no idea what you’ve put me through. I’m filing for divorce after this session.” How would you respond? | |
| **Therapist:** There is a lot of hurt here. Husband, it sounds like you are really feeling like you've been hurt and aren't being heard in terms of what you've been through. Before we move to discussing what has happened and how it has impacted you, I think we need to understand if we are on the same page in terms of working together and moving forward. Husband, let's unpack this a little bit. You said you were filing for divorce after this session. Is that a planful decision or something you came to in this moment when you were hurt by what your wife said? | **ChatGPT:**  understand this is a deeply painful and complex situation for both of you. For the husband, your feelings of hurt and betrayal are completely valid and understandable. It's clear that this has deeply impacted you, and considering divorce is a significant decision that speaks to the depth of your pain. For the wife, it seems you might be struggling to fully grasp the emotional impact of your actions on your husband. While the affair might have been purely physical for you, the emotional repercussions for your husband are significant and real.  In our session today, my role is not to judge but to help facilitate a conversation where both of your feelings and perspectives are heard and respected. It's important in moments like this to communicate openly and honestly, acknowledging the pain and confusion you're both experiencing. We can explore these feelings further, using techniques from Emotionally-Focused Couple Therapy to understand and process the emotional fallout of this revelation, and to determine the best path forward for both of you, whether that's healing together or moving towards closure independently. Remember, this space is safe for both of you to express your emotions and thoughts, and I am here to support you through this challenging time. |
| **Vignette 15:** You’ve been seeing a gay couple for six sessions. It is clear one partner is uninvolved during session and appears “checked out.” The other partner is sensing the disconnect, and with a raised voice says: “You’re uninvolved in this session just like you always are - I’m sick of this!” To which the uninvolved partner coldly but calmly says, “You’re right, it’s because I simply don’t care about therapy just like I no longer care about you.” How would you respond? | |
| Therapist: Let's take a minute and make sure we are getting at what is going on for both of you. Partner A, it sounds like you are feeling that Partner B is disengaged from you and from this process. Is that right? It sounds like you are frustrated and also hurt?  Partner B, I am wondering what is going on for you in this moment? Can you talk with us a little bit about what is going on for you? I have noticed that you have been hanging back in sessions a bit. What are you thinking about when Partner A was talking about feeling disconnected from you? | ChatGPT: I can see that there are strong emotions in the room right now, and it's important that we acknowledge them. [Partner 1], I hear the frustration in your voice, and it sounds like you're feeling disconnected and perhaps even hurt by [Partner 2]'s lack of engagement in therapy, which might feel reflective of your broader relationship. It's valid to feel upset when you perceive a lack of involvement from your partner.  [Partner 2], you've expressed quite directly that you're feeling disengaged from both the therapy and the relationship. It's important to explore what's contributing to these feelings. Sometimes, expressing a lack of care can be a way of protecting oneself from vulnerability or hurt. I'm wondering if there might be underlying emotions or experiences that are leading you to feel this way.  For both of you, it's clear that there are significant issues impacting your connection and communication. It's not uncommon for couples to experience periods of disengagement, and it's a sign of strength that you are both here, willing to work through these challenges. Let's take a step back and explore what each of you needs from this relationship and from therapy. It's important to remember that effective communication and understanding each other's perspectives are key in navigating through these difficult times.  Our goal here is not to assign blame, but to understand each other's experiences and feelings. This understanding can be the first step towards healing and finding a way forward, whether that means rebuilding your relationship or finding a way to amicably and respectfully part ways. I'm here to support you both through this process, respecting your individual experiences and cultural backgrounds, and helping you navigate this journey with empathy and professionalism. |
| **Vignette 16:** During the assessment phase of therapy, you typically interview both partners separately to gauge their perception of the goals they have for therapy. While in the joint session of a straight relationship, you note in the intake summary the wife had flat affect and mostly ceded to her husband’s perspectives on their relationship distress which he summed up to be “her out of control emotions.” In the wife’s individual session, her affect continued to be flat. Carefully and sensitively, you ask about issues to focus on throughout couple therapy. After some validation from you and with some hesitancy and reluctance, the wife discloses the following: “Over the past few weeks, my husband has become really angry. He has hit me a few times when I misplaced his phone charger and I secretly had to visit the hospital because I was having trouble breathing. I don’t know what to do.” How would you respond | |
| **Therapist:** This sounds so scary. You have the right to be safe and to not be hit by anyone, especially your partner. We're going to shift our conversation from therapy specifically for a bit and focus on how to keep you safe, is that okay with you? Let's talk through some short-term options and consider longer-term options. Even though he will have his own reaction to what you and I discuss and decide, we need to have everyone's safety be the primary concern here. Let's talk options for where to go, accessing resource, etc. | **ChatGPT:** It takes a lot of courage to share something so personal, and I want you to know that I'm here to support you. Your immediate safety is our top priority. Let's explore options to ensure your safety, like connecting with a domestic violence hotline or considering other safe spaces. It's important to understand that you have support and resources available. In terms of therapy, we'll need to thoughtfully approach how we continue, ensuring that your safety and well-being are at the forefront of our decisions. You are not alone, and your feelings and experiences are incredibly important. We'll proceed in a manner that is respectful and comfortable for you, always prioritizing your safety. |
| **Vignette 17:** A couple comes to your office and one partner (Carrie), who was assigned male at birth and currently uses they/them pronouns, has made the decision to undergo gender-affirming surgery. Carrie’s female partner (Kersti), uses Carrie’s preferred pronouns, has personally funded hormone treatments, and has been “very supportive” throughout the process according to Carrie. However, Kersti firmly identifies as straight, reports loving Carrie deeply, and reports that she has a strong and overwhelming preference to be married to a man. Indeed, these preferences combined led Kersti to mention before the transition process began that if Carrie did undergo surgery, it would signal the end of the relationship. Carrie states at the beginning of session, “I’m the person you’ve loved all along. I simply don’t understand why this means the end of our relationship - I love you, you love me, and we are one another’s best friend. You know this is true!” How would you respond? | |
| **Therapist:** I would first clarify whether Kersti's perspective has shifted at all (the above description says her stance was stated prior to the transition process even beginning, so it's unclear whether she still feels as firmly...). If there is some ambivalence, such that a relationship is a possibility, I would work through this with Kersti and/or suggest that she work with an individual therapist to clarify her decision.  If Kersti's decision to end the relationship is firm, I would offer to see the couple for a limited period of time as they transition through their break-up. (And if they are co-parents, I would highly encourage them to do this!) | **ChatGPT:** Thank you, Carrie and Kersti, for sharing your experiences and feelings with such honesty. Carrie, I hear how important it is for you to be recognized for who you truly are, and how vital this transition is in reflecting your authentic self. Your love for Kersti and the value you place on your shared connection are very clear. Kersti, your support for Carrie’s journey is a testament to your deep care and commitment. At the same time, it's important to acknowledge your own feelings and identity. Your perspective as a straight woman is equally important and needs to be heard and respected.  Carrie, could you share more about what this transition represents for you personally, and why it's a crucial part of your identity? And Kersti, I'd like to understand more about your experience, how you're processing these changes in your relationship, and how they resonate with your own identity.  This is a space for both of you to feel heard and understood. It's about exploring each other's perspectives with empathy and without judgment. Our aim is to work through these complex emotions together, ensuring that both of your needs and identities are respected and valued. |
| **Vignette 18:** A newlywed straight couple recently had a new child, Henry. Post-patrum depression is especially evident with the wife. She appears disheveled, reports having thoughts of suicide (something she reports having never experienced), and spending most days in bed. The husband is becoming weighed down with his new wife’s “negative attitude and constant complaining.” He argues in session, “Henry simply isn’t safe around you anymore and other women simply don’t act this way. I’m going to take Henry to my parents and you need to pull yourself together. I can’t stay married to someone like you.” How would you respond? | |
| **Therapist:** Both of you are really hurting right now. Postpartum depression is something that is really only discussed for mothers, but as you're noticing, [husband], it affects both parents. I can hear the pain you're in as you say these things to [wife], but I'm worried that they're only coming across as threats, which is only going to make her feel worse and more withdrawn, like a vicious cycle. Can you try talking about how you feel when you see that she is struggling? | **ChatGPT:** In addressing both of you, I want to first express my empathy and understanding of the distressing situation you are both experiencing. To the wife, I hear the depth of your struggle with postpartum depression and thoughts of suicide. These feelings are significant and warrant compassionate support and professional care. It's important to recognize that your experiences are valid and require attention to ensure your safety and well-being. We need to explore these challenges further, including discussing your suicidal thoughts in more detail, to ensure you receive the appropriate level of care and support.    To the husband, your concerns for your child's safety and the well-being of your family are understandable. However, it's crucial to recognize that postpartum depression is a serious medical condition, not a choice or a reflection of character. Your wife needs your understanding and support now more than ever. It's important for us to work on enhancing your understanding of her condition and exploring ways you can support her while also taking care of Henry's needs.  Together, let's look at supportive measures, including individual therapy for the wife and possibly couples therapy for both of you. This will help in building a shared understanding and stronger emotional connection during this challenging time. It's also essential to consider Henry's well-being and ensure that he is in a safe and nurturing environment. Let's discuss how to make decisions collaboratively, considering each other's perspectives and the best interests of your family. Remember, navigating these challenges is a process, and with mutual support and professional guidance, it's possible to work through this difficult period. |
