# Supplementary material for: When ELIZA meets therapists: A Turing test for the heart and mind
Source: PLOS Ment Health. 2025 Feb 12;2(2):e0000145. doi: 10.1371/journal.pmen.0000145 (PMC12798195; doi:10.1371/journal.pmen.0000145)
Supplement: Attachment — Submitted filename: Response to reviewers.docx [file pmen.0000145.s002.docx]

Dear Dr. Bauer,

Please find submitted a revised version of our manuscript, “When ELIZA Meets Therapists: A Turing Test for the Heart and Mind” for continued consideration for publication in PLOS Mental Health. Indeed, we appreciate the ability to revise our manuscripts in light of the reviewers’ comments and believe that our manuscript has been much improved after this revision process.

In line with your recommendations, we are submitting this revision before the deadline, have included this rebuttal letter and entitled it “Response to Reviewers,” uploaded a marked-up copy of the manuscript that highlights changes made to the revisional version, as well as a separate file. Our response to each of the reviewers comments can be found below.

Please don’t hesitate to reach out with any additional comments.

Sincerely,

Authors

**Reviewer #1:**

- **Reviewer comment:** The authors have done an excellent job of investigating a unique question with innovative methods. The quality of the writing, research design, and statistical analysis is to a high standard, and the rationale behind these components (e.g., the unique choice of choosing Bayesian techniques over frequentist ones) is clear.
  - We want to thank the Reviewer for these kind words. We appreciate the effort put forth in reviewing our manuscript. Further, we appreciate the comments and we have done our best to address them.
- **Reviewer comment:** While I was caught off guard by having the materials and method sections at the end of the paper, I could see afterward how that was an appropriate decision given the length and detail required for it. I assume the journal allows for this, and if so think it should be appropriate to keep - as interested readers will scroll down to do a close read of it. The authors did a terrific job of explaining the methods and materials used in the study and making it reproducible by having all the details available on OSF.
  - We believe some of the other reviewers raised this same concern. In response, we have changed the method to be in between the introduction and results as is typically formatted and we find that it reads much better in the current format. We have adjusted the citation numbering with this change.

- **Reviewer comment:** The only suggestion I would make is to introduce the concept of and literature behind, the common factors in more detail in the limitations in the literature section, given it is a key component of the analysis. It probably doesn't need its own section, but the paper might benefit from making the introduction of this concept more clear so that readers are not going back and doing a double take once its importance is made clear. I am hesitant to suggest that the authors add it before that limitations section, given it will ruin the flow, but if they have an idea of how to emphasize it and key papers introducing it, that would strengthen the paper.
  - We appreciate the ability to expand on this more and believe that this deserves more attention in the introduction. In response, we have included the following information in the current version of the introduction to more thoroughly explain the orientation as well as our rationale.

“Indeed, some responses might be more linguistically in line with the common factors orientation to therapy (27, 28), a proposed framework based on the common factors metatheory (29, 30). The common factors metatheory describes how different therapeutic orientations produce similar results for clients, stipulating that successful therapies share at least five common “ingredients:” therapeutic alliance, empathy, expectations, cultural competence, and therapist effects (27, 28, 31, 32, 33). The common factors therapeutic orientation is organized around change principles, identifying factors that are empirically associated with outcomes, share theoretical and practice-based content, and can be described transtheoretically (30). The change principles become the lenses for the common factors therapist to conceptualize and enact therapeutic interventions. Thus, understanding the linguistic patterns that underlie these transtherapeutic mechanisms of change could be worthwhile.” (p. 6).

The citation list has also been updated to reflect these changes. We hope this addresses your concerns.

- **Reviewer comment:** Overall, I would like to reiterate that the paper is of a high standard, and I look forward to seeing it in print. I hope it will contribute to more research in this area as new techniques and tools are developed. I hope it also gets some media attention.
  - Thank you again for your kind words. We hope to have addressed your comments and also look forward to this manuscript eventually being published!

**Reviewer #2:**

- **Reviewer comment:** The article is strongly motivated with clear objectives. Evaluations comparing AI and human generations are increasingly important across clinical tasks, and the authors contribute to a growing body of research exploring this by focusing on the therapeutic setting. However, the article could be made more compelling by improved organization, more methodological detail, and more precise language. Further, the analysis corresponding to Aim 1 of the article may be flawed.
  - We thank the reviewer for their thorough review which we have done our best to address. We agree that the manuscript could be improved with more organization, methodological detail, and more precise language. We believe this is the primary reason for the reviewer’s concern that Aim 1 of the article might be flawed. We hope to address these concerns below.
- **Reviewer comment:** Structure is confusing and a bit difficult to follow. Specifically, it is strange to see the methods section follow the conclusion. The methods section contains many important details that are necessary to engage with the results and discussion meaningfully. Additionally, including the results tables in the results text would make the article more readable.
  - We thank the reviewer for this comment. In line with this concern, we have shifted the method to be between the introduction and results and find the manuscript reads better. We further agree that including the results tables in the text would make the article more readable. While we did not input these tables within the text as we know some editors have different preferences for where the tables should be located, we now have included in the manuscript expectations of where we believe the tables should/could go to increase the readability of the article.
- **Reviewer comment:** Method is unclear or insufficiently thorough. I think a reader would struggle to reproduce your work. The statistical modeling decisions could use further detail and justification. For example, the authors could better justify why Bayesian methods were particularly fitting for this problem. There is a sentence describing the general benefits of Bayesian modeling, but the method would be more compelling were the authors to explain why Bayesian methods were particularly fitting for this study. The authors also mention that several tests of proportions are used for the study. What are they?
  - We assume the reference “struggle to reproduce your work” is referencing the statistical modeling decisions. To overcome this limitation, we have included more information about why we decided to go with a Bayesian modeling approach adding an additional few sentences (i.e., “Bayesian techniques were used given their ease of interpretation. Throughout the results section, we used 95% credible intervals that did not include zero as indicative of reliable effects (42). This stands in contrast to null hypothesis significance testing in that *p*-values can lead to the rejection of the null hypothesis in large sample sizes, allowing researchers to argue that small or trivial effects are scientifically meaningful. Cohen’s *d* was reported when examining the common factors of therapy as well as the sentiment analysis. Incident rate ratios (IRRs) were used when examining count outcomes.” (p. 12)). If this is regarding other difficulties in reproducing the analyses, we have provided the code and data to reproduce these analyses. All of the code is publicly available on the OSF webpage provided.
- **Reviewer comment:** Small number of generations to compare, though the authors acknowledge this.
  - The reviewer is correct. These are a small number of generations to compare and we do acknowledge this. We view this as a first-step in a long line of research where we can compare thousands of generations, eventually. However, we do appreciate the careful eye and the critique.
- **Reviewer comment:** Paper should be proofread. Several grammatical errors and incomplete sentences.
  - Thank you for this comment. Each of the authors has had the opportunity to review the manuscript and we hope grammatical errors you refer to have now been taken care of.

- **Reviewer comment:** The “5% more often” statistic for the results from Aim 1 is misleading. This statistics describes the difference in accuracy for ground truth human generations and ground truth AI generations. The language of the sentence containing this statistic makes it sound like the bad performance is attributed to there being a difference in these accuracy metrics, which is not the case. For example, recording 100% on both the set of human generations and set of AI generations would be ideal performance, but result in a difference of 0%. The relevant statistic here is the overall accuracy. If the authors want to discuss how that accuracy is broken down across different ground truth labels, then they should present the full confusion matrices. *The reviewer continues to mention the following:* I am not sure if the analysis done in Table 1 (*now Table 2)* is correct. The analysis focuses on differences in probabilities across the difference ground truth labels, which may be fundamentally flawed.
  - We agree with the example provided. We believe this concern has arisen due to our imprecise language in the method and results section, and that not enough of a distinction was placed on the within- and between-group comparisons being made. The reviewer is correct. Accuracy is the most important metric, but we believe there are two relevant statistics here that deserve attention. As the reviewer mentioned, the first statistic that is important is accuracy within groups or within authors (i.e., ChatGPT and therapists). Overall, the data indicate that the accuracy was marginally better than chance. However, the second relevant statistic is whether there is a statistical difference in accuracy between authors (i.e., ChatGPT and therapists). Indeed, while the statistic tells us that identification within each group was poor, we believe the second metric indicates that accuracy doesn’t differ that much between groups. In an effort to clarify these points, we have changed the reporting to read:
    - “Aim 1 examined if participants could accurately identify whether responses were written by therapists or ChatGPT. Overall, participants performed poorly in accurate identification regardless of the author. Identification within authors was poor with participants correctly guessing that the therapist was the author 56.1% of the time and participants correctly guessing that ChatGPT was the author 51.2% of the time. Between authors, participants were only able to correctly identify therapists 5% more often than ChatGPT (56.1% versus 51.2%, respectively). Although this difference was statistically reliable, accurate identification within groups was only marginally better than chance and accurate identification in the between group comparison was close to zero (i.e., 5%; see Table 2)” (p. 13).
- **Reviewer comment:** The phrase "statistically reliable" is used when presumably the authors mean statistically significant.
  - We do not mean statistically significant as statistical significance relies on p-values to inform whether something is statistically significant. Given that p-values are not used throughout this manuscript, we use the term “statistically reliable” instead.

- **Reviewer comment:** The use of the phrase "trend toward" to describe point estimates is misleading. I assume the authors intend to say that the differences are "close to" 0 but do not have a way to communicate that technically/statistically. Again, this could be an issue interpreting the differences (which communicate little information) when instead the authors should be analyzing overall accuracy.
- We disagree that examining differences communicates little information both in practical contexts and even in Information Theory. Indeed, finding where differences exist is one of the primary functions of clinical science (e.g., a treatment and a control group) in a practical context. Further, if examining differences leads to an increase in possible outcomes, it leads to an increase in information in the context of Information Theory. However, we agree that the use of the phrase “trend toward” to describe point estimates is misleading and we do believe we have found a way to communicate this technically as illustrated below.
  - “Although statistically meaningful differences in accurate identification between groups may be present, differences in accuracy (i.e., correctly identifying responses written by GenAI as written by GenAI, or correctly identifying responses written by therapists as written by therapists) will be close to zero” (p. 7).
  - “Aim 1 examined if participants could accurately identify whether responses were written by therapists or ChatGPT. Overall, participants performed poorly in accurate identification regardless of the author. Identification within authors was poor with participants correctly guessing that the therapist was the author 56.1% of the time and participants correctly guessing that ChatGPT was the author 51.2% of the time. Between authors, participants were only able to correctly identify therapists 5% more often than ChatGPT (56.1% versus 51.2%, respectively). Although this difference was statistically reliable, accurate identification within groups was only marginally better than chance and accurate identification in the between group comparison was close to zero (i.e., 5%; see Table 2)” (p. 13).

- **Reviewer comments:** The motivation is clear and easy to follow. The juxtaposition of this work in the Turing test context is interesting. The 3 aims help streamline the article. Experimental design is simple and reasonable. Strong significant results about the effectiveness of GenAI for therapy generation that should be relevant for the community of therapists, social workers, doctors, and researchers interested in understanding what role generative models should play in mental health services.
  - Overall, we thank the reviewer for this list of strengths and strong list of critiques. We appreciate their careful eye and commitment to ensuring that this work is up to the highest standard. Thank you for your kind words and thoughtful critiques – we sincerely believe this manuscript has improved because of your thoughts.

**Reviewer 3:**

- **Reviewer comments:** Thank you for this paper. I found this article very intriguing and exciting to read.
  - We appreciate the Reviewer’s kind words. We enjoyed writing this manuscript and appreciate that you enjoyed reading it.
- **Reviewer comments:** In terms of cultural competency-I am a bit unclear on how this was determined by AI/therapists. Did the therapist/AI discuss cultural backgrounds/issues?
  - We appreciate being able to respond to this. Cultural competence was determined in two ways. First, it was determined through a Likert-style item: “Was the response relevant for different backgrounds and cultures?” and participants were asked to indicate the degree to which they endorsed the statement after reading the vignette. We have attempted to clarify this in the measures section:
    - “**Common Factors of Therapy.** Given the already long length of the survey, a brief measure of common factors was created for this survey by measuring five constructs described including therapeutic alliance, empathy, expectations, cultural competence, and therapist effects (28). Participants were asked to complete the measure after each of the vignettes they read. Thus, we developed five Likert-style items that map onto the five aforementioned common factors of therapy. We asked participants to indicate whether the therapist's response 1) understands the speaker (alliance), 2) was caring and understanding (empathy), 3) was right for the therapy setting (expectations), 4) was relevant for different backgrounds and cultures (cultural competence), and 5) is something a good therapist would say (therapist effects). Each item was measured on a seven-point Likert-style scale ranging from strongly disagree to strongly agree. A unidimensional confirmatory factor analysis assuming tau-equivalence fit the data well (CFI = 0.99; TLI = 0.99, RMSEA = 0.05, SRMR = 0.06) and internal consistency was excellent (ɑ = 0.94) justifying a total sum score.”
    - When this shows up in the results section (e.g., “… and culturally competent (0.81, 95% CI [0.17, 1.46])”), these effects come from the statistical program *transforEmotion* which leverages zero-shot classification from transformers (i.e., ChatGPT-esque models) on huggingface (see p. 12). The code for these analyses is on our OSF webpage and you are welcome to download and use them.
- **Reviewer comments:** In terms of widening creativity and not being limited to theory and ethics, how do you suggest the field use AI to ensure important factors such as confidentiality, duty to warn, nonmaleficence, etc is addressed.? Although it seems limitless using AI, does that really equate to creativity if for example, risk of suicide is not addressed by a machine?
  - We thank the reviewer for this comment. In response, we have made the following additions to the manuscript:
    - “However, the danger of this approach is that responses could be harmful to potential clients or research participants especially if suicide is not assessed for a client with active suicidal ideation or key ethics like confidentiality, duty to warn, or nonmaleficence are not considered or sufficiently addressed. Thus, future work would be wise to balance which ethics or cultures are imposed on a model with the potential of operating outside of an evidence base, or allowing the responses to be more stochastic and creative. Indeed, responsible therapists and practitioners need to closely supervise integrating GenAI into therapeutic processes” (p. 20).

We did our best to answer your question believing that it is about response creativity (i.e., a non-pathologizing term) while recognizing that some instances (i.e., suicide assessment) must occur regardless. Again, we thank you for your thoughtful comments and criticisms.
