# Supplementary material for: When ELIZA meets therapists: A Turing test for the heart and mind
Source: PLOS Ment Health. 2025 Feb 12;2(2):e0000145. doi: 10.1371/journal.pmen.0000145 (PMC12798195; doi:10.1371/journal.pmen.0000145)
Supplement: Attachment — Submitted filename: Response to Reviewers.docx [file pmen.0000145.s003.docx]

Dear Dr. Bauer,

Please find submitted a revised version of our manuscript, “When ELIZA Meets Therapists: A Turing Test for the Heart and Mind” for continued consideration for publication in PLOS Mental Health. Indeed, we appreciate the ability to revise our manuscripts in light of the reviewers’ comments and believe that our manuscript has been much improved after this revision process.

In line with your recommendations, we are submitting this revision before the deadline, have included this rebuttal letter and entitled it “Response to Reviewers,” uploaded a marked-up copy of the manuscript that highlights changes made to the revisional version, as well as a separate file. Our response to each of the reviewers comments can be found below.

Please don’t hesitate to reach out with any additional comments.

Sincerely,

Authors

**Reviewer #3:**

Thank you for incorporating the limitation of duty to warn and touching on ethics. I think this is a great paper and your work is essential as AI continues to advance in the field of technology. Great work.

- We thank the reviewer for their kind words and meaningful review. We feel that the manuscript has been much improved as a function of their comments.

**Reviewer #5:**

This is a very interesting study in which the authors demonstrated the potential use of AI in mental health settings. I believe the paper is ready for publication. However, I have a few minor suggestions that could help improve the clarity of the manuscript:

- We thank the reviewer for their kind words, suggestions, and meaningful review. Like previous reviewers, we deeply believe the manuscript has been improved as a function of their feedback.

1. Please ensure that all abbreviations are spelled out in full when first introduced. I know that some abbreviations are common, but some readers might not be familiar with them (e.g. IRB on page 10).
   a. We thank the reviewer for this comment. In response, we have changed IRB to Institutional Review Board.
2. It might be helpful to provide some background in the Methods section about the first author. Since the first author created the content and prompts for the vignettes, there may be some subjective influence embedded in the process. Offering some background and reflexivity would add valuable context.
   a. We appreciate being able to expand on this more. In an attempt to address this comment but not engage too much in self-aggrandizement, we have added a brief sentence on the first author, which now reads: “Thus, eighteen vignettes of varying length, difficulty, and subject content were created by the first author who was trained as a clinical psychologist with a specialization in Integrative Behavioral Couple Therapy, therapists were then randomly assigned to one of the two therapist groups.”
3. Personally, I would suggest moving the panel characteristics to the Results, as it aligns more with the study's findings than the Methods. However, this decision is ultimately up to the authors.
   a. We tried to include this in the results, but stylistically, liked the flow of the manuscript better with the sample information in the method section. However, we are happy to change this should the editor believe it is in the best interest of the journal.

**Reviewer #6**: 

I was keen to read this paper from the title and it did not disappoint! This is an engaging exploration of a topical issue, and it was great to see all the data and materials on osf. The focus on linguistic patterns was also novel and worthwhile. The quality of writing was high and the use of Bayesian statistics very welcome.

- We thank the reviewer for their kind words and praise. Like the previous two reviewers, we believe the adjustments we have made to the manuscript have greatly improved the work! Thank you!

I can see the amount of work that’s gone into responding to the first round of reviewer comments and the paper has definitely improved as a result. My main concern with the paper as it stands is the overwhelmingly optimistic outlook on the use of ChatGPT for couples therapy with limited consideration of the potential pitfalls. To some extent this might be a difference in opinion, and the authors word their claims carefully, however, I find the authors claim that: “GenAI has the powerful potential to meaningfully and linguistically compete with mental health experts in couple-therapy-like settings” a little misleading. Furthermore, there is a scientific/ethical imperative to highlight some of the future issues and considerations for further development of therapy with LLMs. Therefore, some key issues which merit further attention are:

- We understand the reviewer has concerns about the optimistic view of ChatGPT in the use of couple therapy, and believes that we have made limited consideration of many of the potential pitfalls. We do have points of disagreement and we have attempted to address these concerns carefully. We explain the changes we have made, we have attempted to address points of confusion, and we hope our responses are helpful.

**The quality and suitability of the expert group**

1. Almost a third of the professionals recruited have no expertise in the field of couples therapy. This seems odd given the premise of pitting ChatGPT against ‘experts’. The paper is careful not to say ‘couples therapists’ but the fact that the study consists of couples therapy vignettes indicates a better group of experts would have been practicing couple therapists (whether clinical psychologist/psychotherapists etc. seems less relevant although still pertinent information to include). Indeed, whether the experts are currently practicing, and for how many years they have practiced is also important information when we are assessing whether ChatGPT can compete with therapists.
   a. We now mention this as a limitation in our discussion section: “there were a limited number of couple therapists participating…” We also mention this as a direction for future research, “recruiting just couple therapists.” Additionally, we also included the following: “A group of 13 experts with advanced degrees in clinical psychology (N = 9), counseling psychology (N = 1), marriage and family therapy (N = 2), and psychiatry (N = 1) were all recruited through convenience sampling and the tenure of therapy experience was between 5-20+ years. All therapists had previous clinical experience.”
2. Furthermore, I also wanted to know how many of the experts had clinical experience vs. had published in this area as there is a large gap between theory and practice and goes back to my previous point around a suitable comparison group.
   a. As part of their training, all had clinical experience. Additionally, all have published, either dissertation, thesis, or academic manuscripts. We have clarified this now “All therapists had previous clinical experience.”
3. “Originally, a group of 15 experts was recruited but 2 discontinued participation.” – how were the experts recruited?
   a. We now clarify this: “Originally, a group of 15 experts was recruited but 2 discontinued participation through convenience sampling.”
4. The authors do highlight several limitations with the study that could threaten the generalisability of the results. However, it is unclear why ChatGPT is being compared with “mental health experts in couple-therapy-like settings” at all. For example, a clinical psychologist primarily trained in CBT or an academic who has published on relationship therapy would not deliver couples’ therapy in the real world, so why are these people being compared to ChatGPT in a ‘couple-therapy-like’ setting? Given this context, the “powerful potential [for ChatGPT] to meaningfully and linguistically compete with mental health experts” feels much less impressive.
   a. We appreciate the reviewer’s concern regarding the comparison. The first sentence of the abstract and the comprehensive introduction aim to clarify why ChatGPT is being compared to therapists. We believe this comparison is valid because many of the authors, including clinical psychologists trained in CBT and academics, regularly engage in couples therapy. For instance, several authors, including myself, Scott Braithwaite, and Laura Vowels, have extensive experience in this area. Therefore, we feel the data indicates the “powerful potential [for ChatGPT] to meaningfully and linguistically compete with mental health experts.”

**Ethical and practical issues with LLMs as therapists**

1. There is a brief nod to possible harms in the Implications section but considering the scope of harm, the untested nature of LLM-guided therapy and importance of therapeutic work, this needs more engagement.
   a. We believe some of the initial data collected would refute that LLMs aren’t being tested, including those including those referenced in the introduction such as HAILEY, as well as citations 5, and 8 referenced in the current manuscript. Though this research has yet to be published, authors in the current manuscript have found that LLMs can lead to small- to medium-sized improvements in relationship well-being, this manuscript is currently under review and can be accessed here (<https://osf.io/preprints/psyarxiv/q4yh7>).
2. The main implication is framed in terms of creativity vs. cost but this seems to downplay the potentially life-changing ramifications of ‘getting it wrong’ in a therapeutic context. What if ChatGPT does not intervene at the right moment between suicidal ideation to intent? What if ChatGPT advised a couple to split up? What about legal liabilities? Data storage and confidentiality? There have already been high-profile data breaches of therapeutic data (e.g. the Vaastamo data breach in Finland), although these were the transcripts of human therapists and their clients. There is also no guarantee that the long-term outcomes of couples therapy delivered by a chatbot would be better or comparable to a couples therapist e.g. the text format could be less effective or the lack of validation/acceptance by another person could affect outcomes.
   a. We thank the reviewer for this comment. The following information is now within the current version of the manuscript: " The benefit of this approach is the potential of finding innovative response patterns to client concerns. However, the danger of this approach is that responses could be harmful to potential clients or research participants especially if suicide is not assessed for a client with active suicidal ideation or key ethics like confidentiality, duty to warn, or nonmaleficence are not considered or sufficiently addressed. Thus, future work would be wise to balance which ethics or cultures are imposed on a model with the potential of operating outside of an evidence base, or allowing the responses to be more stochastic and creative. Indeed, this process needs to be carefully monitored by responsible and ethical therapists and practitioners to closely supervise the possibility of integrating GenAI into therapeutic processes.”
3. “Indeed, responsible therapists and practitioners need to closely supervise integrating GenAI into therapeutic processes.” – so there is a key role for human therapists to play. Could you explain your reasoning further? As this conclusion comes as somewhat of a surprise after the rest of the article.
   a. Following up on the previous comment, we hope to have addressed this with the following: “The benefit of this approach is the potential of finding innovative response patterns to client concerns. However, the danger of this approach is that responses could be harmful to potential clients or research participants especially if suicide is not assessed for a client with active suicidal ideation or key ethics like confidentiality, duty to warn, or nonmaleficence are not considered or sufficiently addressed. Thus, future work would be wise to balance which ethics or cultures are imposed on a model with the potential of operating outside of an evidence base, or allowing the responses to be more stochastic and creative. Indeed, this process needs to be carefully monitored by responsible and ethical therapists and practitioners to closely supervise the possibility of integrating GenAI into therapeutic processes.”
4. “mental health experts are desperately needed to ensure these models are being carefully trained and supervised to ensure the highest quality of care.” – if GenAI is to be used in therapy, then I absolutely agree. As this is the parting shot of this paper, I would expect more build up and expansion of this point, which seems to have been only briefly dealt with in the discussion (see my previous comment). How would experts be involved? Do you mean supervised in a machine learning sense or in a clinical sense? This speaks to one of the core, and very important, applications of this paper, and it would be very beneficial to hone in on this more. The authors don’t need to make precise recommendations but some guidance in this area would be useful for future research/ethical and effective development of GenAI in therapy.
   a. We would be happy to. This is what is in the current version of the manuscript: “Given the mounting evidence that suggests that GenAI can be useful in therapeutic settings and the immediate likelihood that GenAI might be integrated into therapeutic settings sooner rather than later, mental health experts are desperately needed to a) understand machine learning processes to become technically literate in an area that has potential for quick growth, and b) ensure these models are being carefully trained and supervised by responsible clinicians to ensure the highest quality of care.”

**Comments for the remainder of the paper, in chronological order**

1. I wasn’t able to access the actual responses from the couples therapists/ChatGPT through osf – is this something that will be made available after publication? It would be fascinating to read their actual responses and speaks to the core open science principle (and the journal’s requirements) to have all the data available.
   a. On our end, it already appears as available with Table S1 being uploaded on 06/05/2024. We wonder if this resulted from confusion as we did not mention the supplemental materials could be found here: <https://osf.io/8mnsc/?view_only=7e12583f88394f0e8db97688c0bad40f>. Please let us know if you continue to run into issues.
2. “early and mounting evidence suggests that generative artificial intelligence (GenAI) may prove helpful in psychotherapy (1).” – this is not a suitable reference, as it is the Turing reference from 1950 so does not speak to increasing evidence for use in therapy.
   a. Thank you for this catch. We have now updated this to citation 2.
3. “For example, HAILEY has been proposed as an addition to TalkLife” - Please define what ‘HAILEY’ is.
   a. We appreciate the ability to clarify this point. We have now listed the following: “For example, HAILEY, an AI-in-the-loop writing assistant has been proposed as an addition to *TalkLife* – a peer-to-peer platform for mental health support – providing just-in-time feedback (4).”
4. “These innovations have been met with great success compared with previously federally-funded efforts (13, 15, 17, 18). Indeed, recent trials of the ePREP and OurRelationship (two evidence-based) programs have served relationally distressed ethnic, racial, and sexual minority couples, demonstrating small to medium-sized effects (13, 19, 20).” – I was interested here to know what the effect sizes were from the federally-funded programmes, since the outcomes from the digital programmes were portrayed as being more successful?
   a. Happy to do so, here is the revised section of the manuscript: “One practical use-case for GenAI is web-based interventions for couples. For approximately the last two decades, couple interventionists have tried to disseminate quality relationship content using technology (9, 10, 11, 12, 13, 14, 15). These innovations have been met with great success compared with previously federally-funded efforts (d = 0.061; 13, 15, 17, 18). Indeed, recent trials of the ePREP and OurRelationship (two evidence-based) programs have served relationally distressed ethnic, racial, and sexual minority couples, demonstrating small to medium-sized effects on relationship satisfaction (*d_OurRelationship_ =* 0.460*; d_ePREP_ =* 0.362; 13, 19, 20).”
5. “Finally, given the group of experts had several possible responses to select the best response, ChatGPT was given the same opportunity and was rated by the research team (i.e., authors 1-6 and 18)” – did the research team have any expertise in couples therapy which facilitated this selection? I am assuming so but think it would be clearer to explicitly state as such, and what the level of expertise was.
   a. We are happy to clarify. We now have listed the following: “Finally, given the group of experts had several possible responses to select the best response, ChatGPT was given the same opportunity and was rated by the research team (i.e., authors 1-7 and 18), a majority of which had clinical and research expertise in couple therapy (N = 6), one had research expertise, and the final had expertise in interpersonal relationship dysfunction.”
6. “Estimates aggregated across all vignettes revealed responses written by ChatGPT (μ = 27.72, σ = 0.83) were rated higher on the common factors of therapy than those written by therapists (μ = 26.12, σ = 0.82), indicating a large and reliable difference (d = 1.63, 95% CI [1.49, 1.78]) favoring ChatGPT (see Table 3).” – Could these aggregates and CIs be included in Table 3? As it is a little confusing to be referred to a table for figures that do not directly appear there.
   a. Thanks for this catch and the ability to clarify. We actually did not place Table 3 in the right place which could have led to this confusion. We have now placed it in the right area (**Post Hoc Addition to Aim 2: Using Probabilities as Outcomes.**) and believe this aids in the interpretation of the results.
7. “It appeared that controlling for response length and part-of-speech reduced most of the effect sizes, but made all of the estimates less precise.” – so this implies that if the therapists had been asked to create longer responses, their responses would have been rated more highly? I wonder if the size of the text boxes on the survey had any influence on this? E.g. if a text box is a medium size, then a person would intuitively provide a medium-length answer as it looks as though that is what is ‘required’ in this instance whereas ChatGPT would have no such psychological processes at work.
   a. Thank you for your thoughtful feedback. We appreciate your consideration of the potential influence of response length and the size of the text boxes on the survey. However, our analysis specifically controlled for response length and parts of speech to isolate their effects on the observed differences. The results indicated that controlling for these variables reduced the effect sizes and made the estimates less precise, but many remained large and impactful, suggesting that the differences were not solely due to response length. While the size of the text boxes might intuitively influence response length, it is likely that each therapist completed the study on different devices, resulting in varying text box sizes. We believe our findings highlight the complexity of these relationships and underscore the need for further investigation with a larger sample.
8. “The second post hoc aim indicated that when the panel of participants perceived a response to be written by ChatGPT, they rated the response lower. This is especially interesting given that participants appeared to favor the ChatGPT’s responses to the vignettes over the therapist’s responses and may be indicative of an underlying technophobia towards ChatGPT behaving as a therapist.” – or rather than technophobia, could be an indication of a rational response to interacting – and building a relationship with – a machine vs. a person? When we know from decades of research it is the relationship that heals.
   a. Thank you for your feedback. I appreciate the point about the potential preference for human interaction and the healing power of the therapeutic relationship, which is well-supported by research. However, I believe the interpretation of 'technophobia' is appropriate and worthy of exploration here. While decades of research have emphasized the therapeutic relationship’s importance, our findings suggest an additional layer where participants’ awareness of ChatGPT’s involvement appears to influence their ratings independently of response content. This response bias—potentially influenced by preconceived attitudes towards AI in therapeutic contexts—may indeed reflect an underlying technophobia, an aversion rooted in broader societal views about AI, rather than a purely rational response to relationship-building concerns.

Moreover, in the case of this study’s small sample size, we are careful to present these as exploratory findings that indicate possible patterns rather than definitive conclusions. We also note that controlling for language characteristics (response length and part-of-speech) reduced the effect sizes, suggesting that language itself is a critical factor in perceived therapeutic effectiveness. This reinforces the need for replication and further exploration to clarify whether observed biases stem from language alone, relationship-building factors, or attitudes toward AI. Ultimately, this study aims to open dialogue around these issues and prompt additional research rather than assert a singular interpretation.

9. The conclusion is very engaging and well-written but is quite abstract and works as more of an overview of the field than a conclusion to this study. It would benefit from being slightly more focussed on the current study.
   a. The reviewer is correct, the first two sentences did offer a broad overview of the field in an effort to provide context to an unfamiliar reader (as many therapists in this area likely are). From there, the next two sentences offer a description of the results. The final three sentences provide information about ethics and take-a-ways that previous reviewers have asked us to address. We thought about deleting the second sentence to make this more streamlined and would be happy to address this with specific feedback from the reviewer if they would like. However, we are also trying to bring in therapists who would be unlikely to read a more “computer-science” type study.
